# Past, Present, and Future of Phosphate Management

**DOI:** 10.1016/j.ekir.2022.01.1055

**Published:** 2022-02-01

**Authors:** Simit M. Doshi, Jay B. Wish

**Affiliations:** 1 Division of Nephrology, Department of Medicine, Indiana University School of Medicine, Indianapolis, Indiana, USA

**Keywords:** chronic kidney disease, end stage renal disease, hyperphosphatemia, phosphate binder, phosphate management, serum phosphorus

## Abstract

Cardiovascular (CV) disease (CVD) accounts for >50% of deaths with known causes in patients on dialysis. Elevated serum phosphorus levels are an important nontraditional risk factor for bone mineral disease and CVD in patients with chronic kidney disease (CKD). Given that phosphorus concentrations drive other disorders associated with increased CV risk (e.g., endothelial dysfunction, vascular calcification, fibroblast growth factor-23, parathyroid hormone), phosphate is a logical target to improve CV health. Phosphate binders are the only pharmacologic treatment approved for hyperphosphatemia. Although their safety has improved since inception, the mechanism of action leads to characteristics that make ingestion difficult and unpleasant; large pill size, objectionable taste, and multiple pills required for each meal and snack make phosphate binders a burden. Side effects, especially those affecting the gastrointestinal (GI) system, are common with binders, often leading to treatment discontinuation. The presence of “hidden” phosphates in processed foods and certain medications makes phosphate management even more challenging. Owing to these significant issues, most patients on dialysis are not consistently achieving and maintaining target phosphorus concentrations of <5.5 mg/dl, let alone more normal levels of <4.5 mg/dl, indicating novel approaches to improve phosphate management and CV health are needed. Several new nonbinder therapies that target intestinal phosphate absorption pathways have been developed. These include EOS789, which acts on the transcellular pathway, and tenapanor, which targets the dominant paracellular pathway. As observational evidence has established a strong association between phosphorus concentration and clinical outcomes, such as mortality, phosphate is an important target for improving the health of patients with CKD and end-stage kidney disease (ESKD).

CVD is a primary contributor to mortality in patients with CKD and ESKD, accounting for more than half of deaths with known causes in patients on dialysis.^1^ The prevalence of CVD has not decreased over time: the prevalence of CVD in patients with CKD was 69% in 2013^2^ and 63% to 75% (depending on disease stage) in 2018,^1^ whereas the prevalence of CVD in patients on dialysis was 61% to 74% in 2013^2^ and 65% to 77% in 2018.^1^ Mortality owing to CVD in patients with ESKD has increased, with CVD accounting for 51% of deaths with known causes in patients with ESKD in 2011 to 2013^2^ and 53% and 55% of deaths with a known cause in patients on peritoneal and hemodialysis, respectively, in 2018.^1^ These data indicate that novel approaches to improving CV health in patients with CKD and ESKD are needed.

The complexity of maintaining CV health in patients with CKD is high because nontraditional factors also increase the risk of CVD in this population; in addition to traditional risk factors (e.g., smoking, diabetes, hypertension),^3^ mineral and endocrine abnormalities are key nontraditional risk factors associated with increased CV morbidity and all-cause mortality.^4^ A meta-analysis of CV events in patients with CKD explored the association between CV health and nontraditional risk factors, including serum phosphorus, albumin, hemoglobin, and urate.^3^ This study found that increased serum phosphorus concentrations were associated with increased risk of CV events (pooled hazard ratio 1.20 per mg/dl increase, 95% CI 1.08–1.33, *P* = 0.005).^3^ Nevertheless, most patients on dialysis are not consistently achieving and maintaining target phosphorus goals of <5.5 mg/dl, let alone more normal levels of <4.5 mg/dl. Furthermore, there was no major change in the proportion of patients with all mineral bone disease markers (serum phosphorus, calcium, and parathyroid hormone) within the recommended range from 2010 (31%) to 2021 (30%).^5^

Improving phosphate management is a rational approach to improving CV health because phosphate retention and elevated phosphorus concentrations trigger multiple pathophysiological derangements associated with increased risk of CVD. High phosphate level leads to endothelial dysfunction, causing cell injury by inducing endothelial cell apoptosis and disrupting mitochondrial function by increased production of reactive oxygen species.^6^ It also induces the calcification of vascular smooth muscle cells,^7^ which increases the risk of CV and all-cause mortality.^8^ Phosphate retention triggers increases in fibroblast growth factor-23 and parathyroid hormone concentrations, which are both linked to CV morbidity and mortality. Elevated fibroblast growth factor-23 concentrations are associated with congestive heart failure^9^ and induce left ventricular hypertrophy.^10^ High parathyroid hormone concentrations are associated with hypertension^11^ and increased risk of CV mortality.^12^ Improved phosphate management could remove or reduce the stimulus for all these abnormalities, potentially decreasing the risk of CVD.

### Evolution of Phosphate Management Therapies

Aluminum salts, introduced in the 1970s, were the first phosphate binders^13^^,^^14^ (Figure 1). Aluminum salts reduce phosphate availability by forming coordination compounds with phosphate ions, creating insoluble aluminum phosphate precipitates in the GI tract.^15^ Although effective, use of aluminum-based phosphate binders was largely discontinued in the 1980s owing to an association with neurotoxicity,^16^ cognitive disturbances, osteomalacia, and anemia.^17^^,^^18^

**Figure 1:**
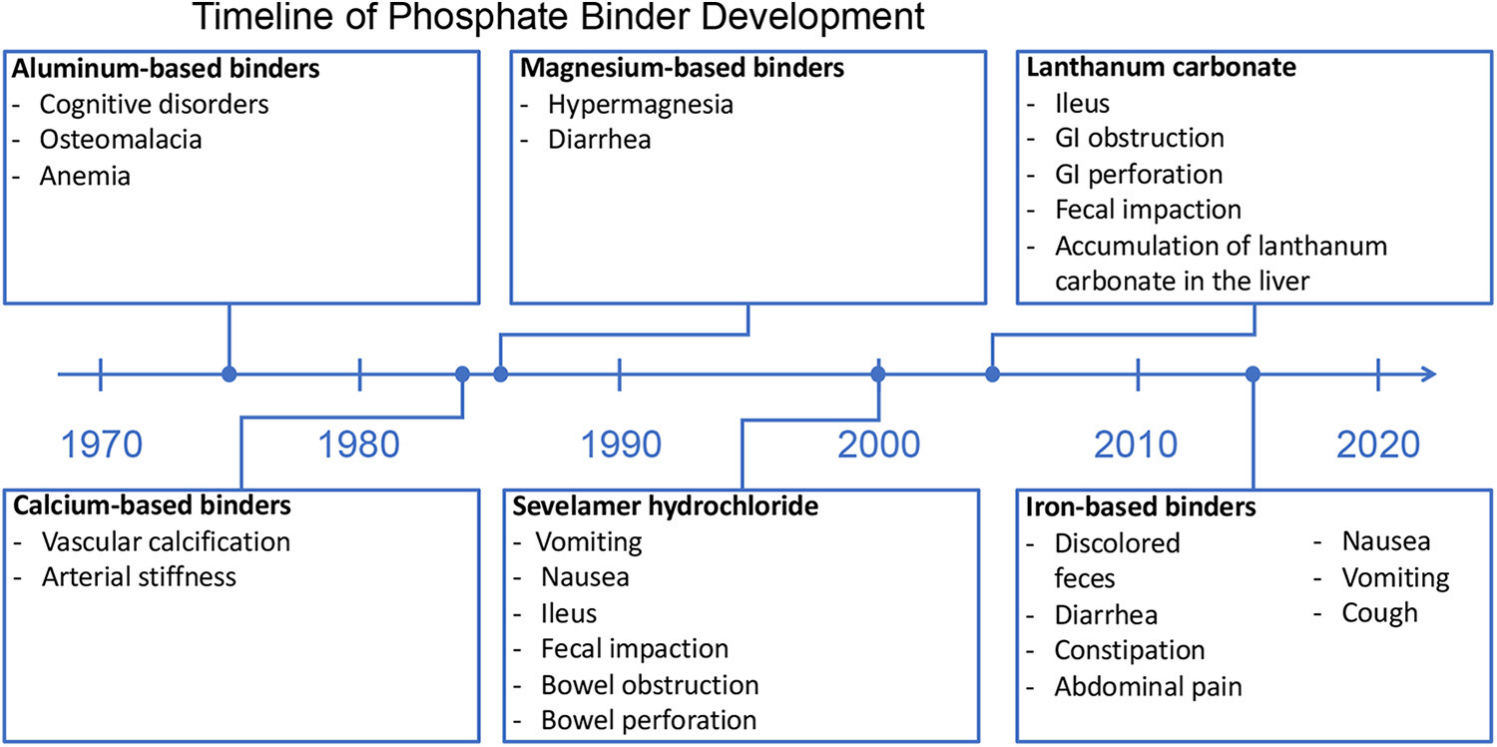
Timeline of phosphate binder development. Phosphate binders were first introduced in the 1970s. Severe safety concerns (e.g., cognitive disorders) were associated with aluminum-based binders, the earliest iteration of phosphate binders. The safety of phosphate binders has improved since their introduction, but adverse effects, particularly those affecting the GI system, are still common for current options. GI, gastrointestinal.

Calcium-based phosphate binders first appeared in the mid-1980s as a potential replacement for aluminum-based phosphate binders.^14^^,^^19^ When first introduced, they were effective,^20^^,^^21^ inexpensive,^22^ and widely used.^13^ Nevertheless, calcium-based binders were soon recognized as potential drivers of vascular calcification and, thus, contributors to increased CV mortality.^23^ Increased calcium load from the use of calcium-based binders has been associated with vascular calcification and increased arterial stiffness.^24^^,^^25^ Recognizing the adverse effects of exogenous calcium intake, the Kidney Disease: Improving Global Outcomes revised its guidelines in 2017 to recommend restricting the dose of calcium-based phosphate binders in adults with CKD stages G3A to G5D.^26^

A combination of magnesium hydroxide and aluminum hydroxide was found to be effective for phosphate control in the 1980s and did not cause uncontrolled hypermagnesemia.^27^ Calcium acetate/magnesium carbonate was also found to effectively lower phosphorus levels. In a study of patients on dialysis, calcium acetate/magnesium carbonate reduced serum phosphorus levels and was not associated with an increased risk of hypercalcemia, although total serum calcium level did increase.^28^ A study of calcium acetate/magnesium carbonate in an animal model evaluated the possible harmful effect of magnesium on bone turnover and mineralization.^29^ Results revealed that calcium acetate/magnesium carbonate at doses that reduced vascular calcification did not adversely affect bone remodeling or alter bone magnesium levels.^29^

Sevelamer hydrochloride was approved by the US Food and Drug Administration in 2000.^30^ Trial data revealed that sevelamer decreased phosphorus concentrations without increasing calcium load.^31^ This is supported by a Cochrane systematic review of phosphate binders, which found in patients on dialysis, sevelamer may lead to lower all-cause death (relative risk = 0.53, CI 0.30–0.91) and induce less hypercalcemia (relative risk = 0.30, CI 0.20–0.43) than calcium-based binders.^32^ This meta-analysis did not reveal any clinically significant difference among phosphate binders for outcomes of CV death, myocardial infarction, stroke, fracture, or coronary artery calcification.^32^ Nevertheless, sevelamer hydrochloride was found to worsen metabolic acidosis^33^ and sevelamer carbonate was developed as an alternative.^34^ One randomized, crossover study comparing sevelamer hydrochloride (800 mg tablets at an equivalent dose to whatever binder each patient was taking before the study) and sevelamer carbonate powder (individualized dose based on each patient’s most recent sevelamer hydrochloride dose) found equivalent phosphorus control, with improved bicarbonate levels during sevelamer carbonate treatment.^35^ These results conflict with those from a separate randomized, parallel study comparing thrice-daily sevelamer hydrochloride tablets and once-daily sevelamer carbonate powder at starting doses of 4.8 g/d, with the option to titrate up or down as needed.^36^ Patients treated with sevelamer hydrochloride had a greater mean decrease in serum phosphorus than those treated with sevelamer carbonate (2.9 mg/dl vs. 2.0 mg/dl), and noninferiority was not found.^36^

Lanthanum carbonate, approved in 2004,^37^ also reduces phosphorus levels without increasing calcium load,^38^^,^^39^ potentially decreasing the risk of treatment-related hypercalcemia (relative risk = 0.16, CI 0.06–0.43).^32^ Nevertheless, the prescribing information for lanthanum includes a precaution that serious cases of GI obstruction, ileus, GI perforation, and fecal impaction have been reported.^37^ Some cases required surgery or hospitalization.^37^ Patients are advised to chew the tablet completely to reduce the risk of these serious adverse GI events.^37^ In addition, accumulation of lanthanum carbonate in the liver has been observed in animal models, and increase of tissue lanthanum content was enhanced in uremic rats in comparison to normal rats.^40^ The liver is the main route for lanthanum excretion, and accumulation in the liver is predominantly noted in lysosomes.^41^ A clinical trial on 2000 patients with ESKD with a median follow-up of 4 years failed to reveal conclusive evidence of hepatotoxicity related to lanthanum (Hutchison A. Analysis of liver function and hepatobiliary adverse event data from 2000 dialysis patients participating in clinical trials on the new phosphate binder, lanthanum carbonate [abstract]. Nephrol Dial Transplant. 2005;20(suppl 5):v93). Hence, preexisting liver disease is not a contraindication to prescribing lanthanum.

Other noncalcium, iron-based binders are sucroferric hydroxide and ferric citrate. Sucroferric hydroxide was approved in 2013.^42^ It effectively reduces phosphorus levels in patients undergoing dialysis and has a lower pill burden than sevelamer carbonate.^43^ Ferric citrate was approved in 2014.^44^ A meta-analysis included 16 studies evaluating ferric citrate use and revealed it to be effective in lowering phosphorus and phosphorus-calcium product versus no active treatment, with comparable efficacy with other phosphate binders.^45^ Nevertheless, the medication caused significantly higher GI side effects.^45^

### Challenges in Achieving Target Phosphorus Concentration

Although phosphate binders have been found to reduce phosphorus concentrations in clinical trials, many patients on dialysis are still unable to achieve target phosphorus concentrations. This may be attributed to an abundance of “hidden” phosphates in food, suboptimal adherence stemming from the burden of taking phosphate binders multiple times per day, and side effects of phosphate binders.

“Hidden” phosphates in food and medications increase phosphate intake and make it more challenging to achieve phosphate control. Phosphate additives are used in many processed foods and are estimated to increase daily phosphate intake by approximately 1000 mg,^46^ resulting in a total daily phosphate intake of up to approximately 2400 mg.^46^^,^^47^ These additives are “hidden” because the quantity of phosphorus they contain is not required to be listed on food labels.^48^ Medications may be another source of hidden phosphate, as phosphate excipients are a common addition to medications prescribed to patients with CKD and can contribute an additional 100 to 200 mg of phosphate per day^49^^,^^50^ (Table 1). These “hidden” phosphate sources increase phosphate load and make it difficult for patients to accurately calculate their daily phosphate intake.

**Table 1:** Phosphate excipients in typically prescribed drugs for patients on dialysis

Generic drug name	Indication	Brand name	Manufacturer	Dose per pill/tablet (mg)	Quantity of phosphate excipients per pill/tablet (mg)
Clonidine hydrochloride	Attention deficit hyperactivity disorder^51^	KAPVAY	Blue Point Laboratories^50^	0.2^51^	1.4^51^
Paroxetine	Major depressive disorder, panic disorder, social anxiety disorder, premenstrual dysphoric disorder^52^	PAXIL CR	GlaxoSmithKline Inc.^50^	40^51^	111.5^51^
		N/A	Cadila Pharmaceuticals^50^	40^51^	22.7^51^
Amlodipine besylate	Hypertension, coronary artery disease^53^	NORVASC	Lupin Pharmaceuticals	10^51^	8.6^51^
		N/A	Greenstone LLC^50^	10^51^	27.8^51^
		N/A	Qualitest Pharmaceuticals^50^	10^51^	40.1^51^
Lisinopril	Hypertension, heart failure, acute myocardial infarction^54^	PRINIVIL	Merck^50^	10^51^	21.4^51^
		N/A	Blue Point Laboratories^50^	10^51^	32.6^51^
Reno Caps	Multivitamin product for patients on dialysis^50^	N/A	Nnodum Pharmaceuticals^50^	N/A^51^	1.7^51^
Renavite	Multivitamin product for patients on dialysis^50^	N/A	Cypress Pharmaceuticals Inc.^50^	N/A^51^	37.7^51^

N/A, not available.

The phosphate binder mechanism of action may contribute to characteristics of these medications that make ingestion difficult and unpleasant for patients. Binders complex with dietary phosphate when both are in the GI tract simultaneously, resulting in nonabsorbable compounds that are excreted.^30^^,^^37^^,^^42^^,^^44^^,^^55^ Because binders have limited phosphate binding capacity per pill,^56^ patients are required to take many pills with each meal and snack.^30^^,^^37^^,^^42^^,^^44^^,^^55^ Patients have also expressed dislike of the large size and bad taste of binders,^57^ and an analysis of reasons for phosphate binder discontinuation found that 8% of patients stopped taking binders owing to an inability to chew/swallow the pills.^58^ All these properties make phosphate binders a burden for patients.

Phosphate binders account for approximately 50% of the total daily pill burden for patients on dialysis.^59^ Chiu *et al.*^59^ found that the median daily count was 9 phosphate binder pills. When broken down by binder type, the median pill burden was 11 for sevelamer monotherapy, 9 for calcium-based binder monotherapy, 6 for lanthanum monotherapy, and 13 for combination therapy.^59^ A separate chart review reported an average daily pill count of 7 for ferric citrate, 11 for sevelamer carbonate, 9 for calcium acetate, and 16 for sevelamer carbonate and calcium acetate.^60^ Chiu *et al.*^59^ also reported that only approximately 40% of patients were adherent to the phosphate binder therapy. A potential explanation for poor adherence is that taking large quantities of pills on a regular basis may be unpleasant for patients. As treatment efficacy depends on proper adherence to labeled dosing instructions, nonadherence may contribute to the inability of many patients on dialysis to achieve and maintain target phosphorus concentrations.

The need to carry phosphate binders at all times may lead to stress and/or anxiety for patients and affect patients’ social interactions. Thus, novel therapies that effectively reduce phosphate to more normal levels and address these burdensome characteristics would likely be welcome.

Side effects of phosphate binders, particularly those affecting the GI system, are common and may lead to treatment discontinuation. An analysis of reasons for phosphate binder discontinuation found that 11% of patients stopped treatment owing to nontolerance, and within this subgroup, 48% discontinued owing to GI upset.^58^ GI side effects such as nausea, vomiting, diarrhea, and constipation are among the most common adverse effects listed on phosphate binder labels (Table 2).

**Table 2:** Gastrointestinal adverse effects on phosphate binder labels

Phosphate binder	Brand name	Adverse effects
Calcium acetate^59^	PhosLo	Nausea Vomiting
Sucroferric oxyhydroxide^42^	VELPHORO	Diarrhea Discolored feces
Lanthanum carbonate^37^	FOSRENOL	Nausea Diarrhea Vomiting Abdominal pain Constipation (postmarketing) Dyspepsia (postmarketing)
Ferric citrate^44^	AURYXIA	Nausea Vomiting Diarrhea Abdominal pain Constipation Discolored feces
Sevelamer carbonate^30^	RENVELA	Nausea Vomiting Diarrhea Abdominal pain Constipation Dyspepsia Flatulence Cases of fecal impaction, ileus, bowel obstruction, and bowel perforation have also been reported

Although patients on dialysis are instructed to limit dietary phosphate intake, they may not understand why it is important to control phosphate or the negative consequences of hyperphosphatemia. It has been found that patients on dialysis have limited knowledge on phosphate compared with other nutrients.^61^ Thus, clinicians should clearly communicate the morbidity and mortality risks associated with hyperphosphatemia. Dietary counseling and education have been found to improve phosphate control,^62^^,^^63^ so increased education may improve outcomes. Clinicians' perception of the importance of taking phosphate binders may also affect patient education: a survey of dialysis providers found that physicians and nurse practitioners believed that it was “less true” that phosphate binders are very important for patients on dialysis, whereas dialysis technicians offered a higher level of support.^64^

Many patients on dialysis are not able to consistently achieve target phosphorus concentrations, and phosphate control has not changed substantially in the past 5 years. The proportion of patients on dialysis with a most recent phosphate level > 5.5 mg/dl was 35% in 2016 and 43% in 2021, and the proportion of patients on dialysis with a most recent phosphate level > 4.5 mg/dl was 67% in 2016 and 71% in 2021. This lack of improvement points to the inadequacy of current phosphate management strategies.

### New Mechanisms for Phosphate Control

The inability of current phosphate management strategies to consistently achieve and maintain target phosphorus levels is concerning,^65^ given the association of hyperphosphatemia with serious negative consequences.^66^ Novel approaches to phosphate management are needed, and innovative therapies should leverage the new understanding of intestinal phosphate absorption pathways.

In the intestine, dietary phosphate is absorbed actively by the saturable transcellular pathway and passively by the nonsaturable paracellular pathway.^67^ Active uptake of phosphate in the transcellular pathway is facilitated by the type II sodium-dependent phosphate cotransporter NaPi2b.^68^ In patients with ESKD, the transcellular pathway was found to saturate above a luminal concentration of approximately 6 mg/dl (2 mM/l).^67^ Calculations based on gastric volume determined that the dietary phosphate intake of up to 2500 mg/d associated with a typical Western diet would result in luminal phosphorus concentrations of approximately 55 to 110 mg/dl (18–36 mmol/l),^46^^,^^47^^,^^69^^,^^70^ far exceeding the maximum amount of phosphorus that can be transported by the transcellular pathway. Studies in humans and animals revealed that passive phosphate absorption by the paracellular pathway occurs along concentration gradients through tight junction complexes (e.g., claudins and occludins) between cell membranes.^71^^,^^72^ Animal data reveal that 65% to 80% of intestinal phosphate absorption occurs paracellularly,^73^^,^^74^ and human data support the dominance of the paracellular pathway, particularly when phosphate concentrations are high^67^ (Figure 2a and 2b).

**Figure 2:**
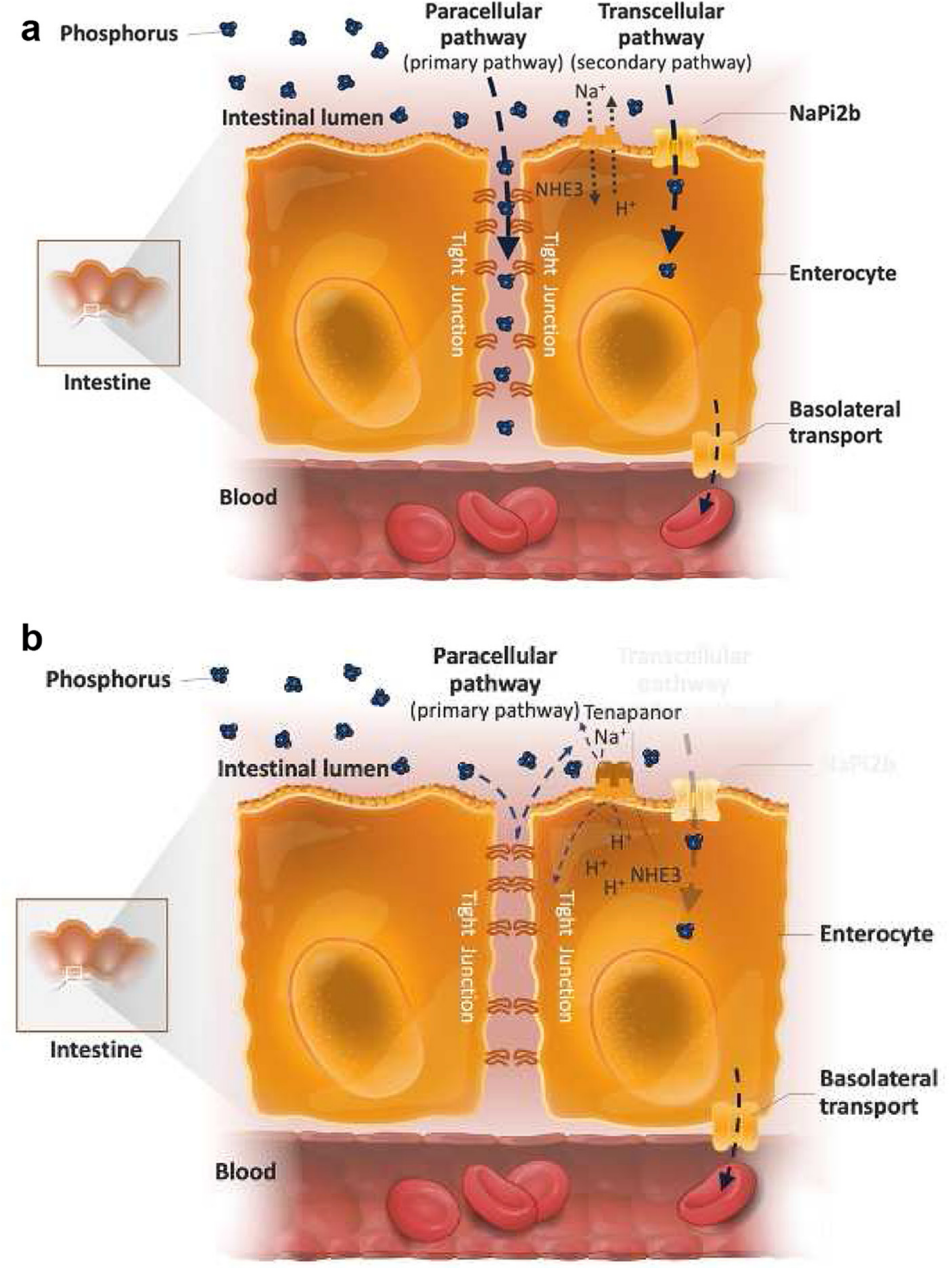
Intestinal phosphate absorption pathways.^67^^,^^68^^,^^72^ (a) Phosphate absorption in the intestines takes place by the transcellular and the paracellular pathways. Phosphate uptake through the secondary transcellular pathway is facilitated by the sodium-dependent phosphate transporter NaPi2b. Passive phosphate diffusion in the dominant paracellular pathway occurs along the concentration gradient through tight junctions. (b) Tenapanor is a nonbinder phosphate control therapy that reduces paracellular phosphate absorption by decreasing tight junction permeability to phosphate.

New therapies targeting intestinal phosphate absorption pathways have been developed. Phase 1 trials of the novel drug EOS789, an inhibitor of the sodium phosphate cotransporter NaPi-2b, PiT-1, and PiT-2, revealed encouraging results in patients receiving hemodialysis.^75^ Fractional phosphate absorption trended lower in patients receiving EOS789 (100 mg) in comparison to those treated with placebo, and the study drug was safe and well-tolerated.^75^ EOS789 was compared with placebo and with/without sevelamer to study an additive benefit. Fractional phosphate absorption was lower in patients treated with 100 mg EOS789 (0.40) than in those treated with placebo (0.53), and patients treated with 100 mg EOS789 with 1600 mg sevelamer (0.36) fared even better than those with EOS789 alone, indicating an additive benefit.^75^ Nicotinamide, which seems to reduce phosphate-specific transcellular permeability by inhibiting gut NaPi2b cotransporters, is another potential hyperphosphatemia treatment.^76^ Nevertheless, no significant reductions in phosphorus were observed in a 12-month trial of nondialysis patients with CKD treated with nicotinamide.^77^ Multiple small clinical trials in patients with ESKD have reported reduction in serum phosphorus concentration, but larger studies are lacking to support widespread use.^78^

Another novel drug that targets a phosphate absorption pathway is tenapanor, an investigational, nonbinder therapy that inhibits the sodium/hydrogen exchanger isoform 3 (NHE3) in the GI tract. Inhibition of NHE3 blocks paracellular phosphate permeability by reducing sodium absorption and causing conformational changes in tight junction proteins.^72^ Tenapanor has been found to efficiently reduce phosphorus levels in multiple clinical trials.^79^^,^^80^ In a comparison of tenapanor plus phosphate binders (“dual-mechanism”) versus placebo plus phosphate binders, patients in the dual-mechanism group achieved a greater mean decrease in serum phosphate concentrations from baseline to week 4 compared with those treated with placebo and phosphate binders (0.84 vs. 0.19 mg/dl, *P* < 0.001).^79^ The most frequently reported adverse event was diarrhea, resulting in study drug discontinuation in 3.4% and 1.7% of patients in the dual-mechanism and placebo plus binder groups, respectively.^79^ A separate long-term study supports the tolerability of tenapanor: rates of serious adverse events were higher in patients treated with sevelamer carbonate (16.4%–23.4%) versus tenapanor (11.2%–17.4%) across all study periods.^81^ No clinically meaningful changes in serum calcium, bicarbonate, chloride, potassium, sodium, or glucose were observed.^80^ It is administered as 1 tablet taken twice a day, which may significantly decrease the pill burden for patients with hyperphosphatemia who currently need to take approximately 9 phosphate binder pills each day.^59^^,^^82^ A recent trial revealed that 72% of patients achieved the primary end point of ≥30% decrease in the number of daily binder and tenapanor tablets compared with the number of daily binder tablets at baseline (*P* < 0.001). The mean total number of phosphate-lowering tablets per day decreased from 15 at baseline to 3 at week 26, with a mean decrease of 12.1 binder tablets per day, and 30% of patients completely switched from binders to tenapanor (*P* < 0.001).

### Observational Data Link Phosphate to Poor Outcomes but Randomized Trial Data Are Lacking

There is abundant observational evidence that links elevated phosphorus with increased risk of mortality and CVD. Block *et al.*^83^ found that serum phosphorus concentrations >5.0 mg/dl were associated with an increased relative risk of death (1.07, 1.25, 1.43, 1.67, and 2.02 for serum phosphorus 5.0–6.0, 6.0–7.0, 7.0–8.0, 8.0–9.0, and ≥9.0 mg/dl, respectively). Kestenbaum *et al.*^84^ reported that each 1 mg/dl increase in phosphorus levels was associated with a 23% increase in mortality risk. Tonelli *et al.*^85^ noted that individuals with serum phosphorus level ≥3.5 mg/dl had an adjusted hazard ratio for mortality of 1.27 compared with those with phosphorus level <3.5 mg/dl. Dhingra *et al.*^86^ found that individuals with phosphorus levels in the highest quartile (3.5–6.2 mg/dl) experienced a multivariable-adjusted 1.55 CVD risk compared with those in the lowest quartile (1.6–2.8 mg/dl).

Conducting prospective, randomized trials with hard clinical end points in patients with CKD, analogous to those that have led to major therapeutic advances in other fields (e.g., oncology and cardiology), is an established challenge.^87^ Given the guideline-recommended target phosphorus level of <5.5 mg/dl, we believe that it would be unethical to conduct a trial evaluating adverse effects of phosphorus level elevated beyond this threshold. In addition, CKD is a multifactorial disease associated with complications spanning different physiological processes, such as CVD,^88^ dyslipidemia,^89^ anemia,^90^ and mineral bone disorder^91^ (Figure 3a and 3b). Nevertheless, the high volume of evidence connecting elevated phosphorus level with CV and overall mortality, and the data indicating phosphate drives multiple physiological changes that increase CV risk, establish phosphorus concentrations as a logical target for intervention in CKD. A lack of resources may also contribute to the dearth of randomized clinical trial data; well-designed, adequately powered randomized trials studying hard outcomes are expensive (e.g., $50–$100 million) and time consuming.^92^^,^^93^

**Figure 3:**
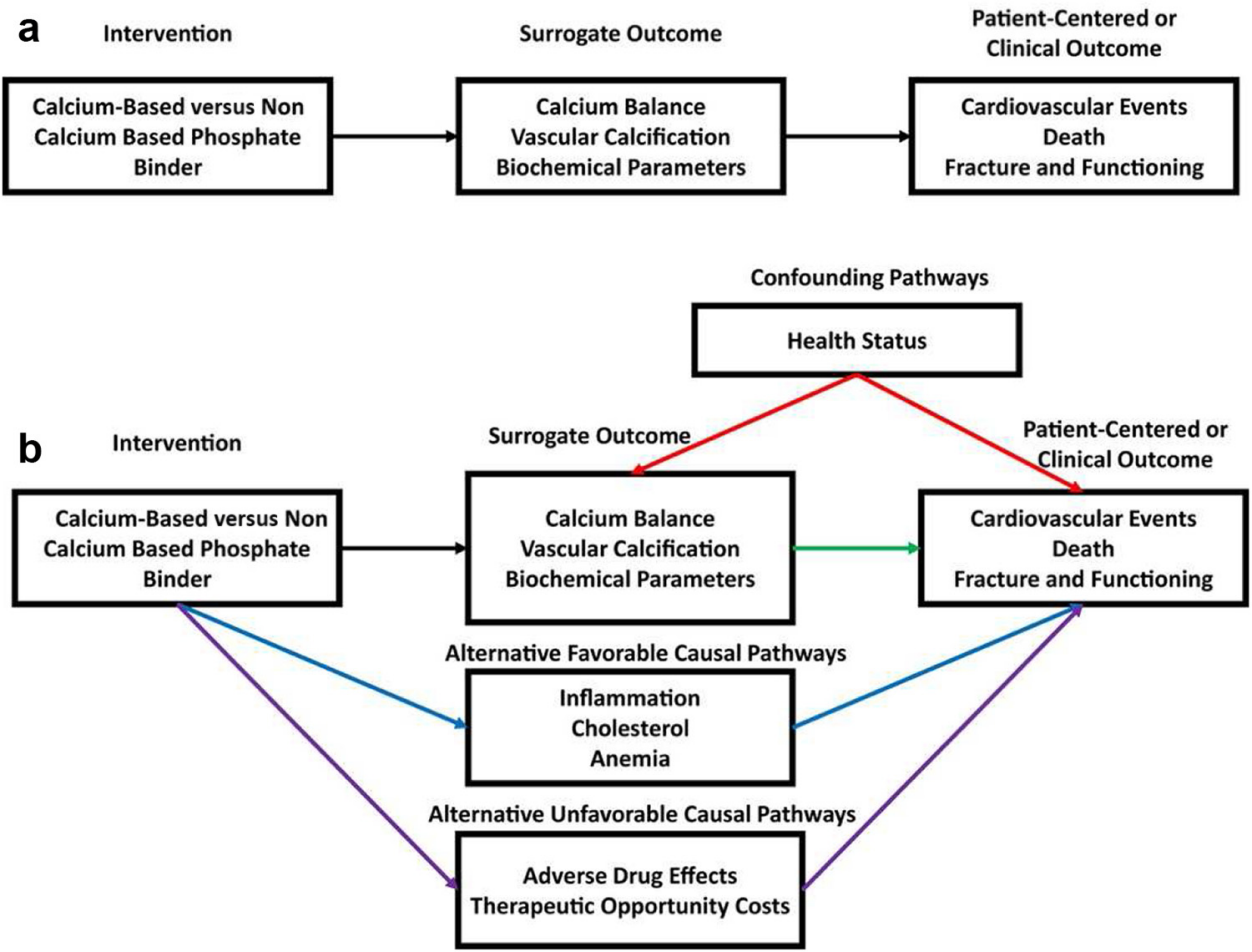
Chronic kidney disease is a multifactorial disease that affects many physiological processes. The multifactorial nature of chronic kidney disease complicates the use of current surrogate outcomes in phosphate binder trials. (a) Ideally, an intervention influences the patient-centered or clinical outcome exclusively through the surrogate outcome. (b) This idealized situation may not be true for phosphate binder trials. Multiple confounding pathways (red arrows) between the surrogate and patient-centered or clinical outcome may induce correlation without causation. In addition, both favorable (blue arrows) and unfavorable (purple arrows) alternative pathways between the surrogate and patient-centered or clinical outcome may mean that the impact of the intervention on the clinical outcome is not fully captured by the surrogate outcomes.

Single-intervention trials evaluate outcomes specific to the intervention, not hard clinical end points. For example, phosphate binders were approved based on their ability to lower phosphorus levels in patients with CKD, not impact on morbidity or mortality.^30^^,^^37^^,^^42^^,^^44^^,^^55^ Single dialysis-based interventions, such as increasing the dialysis dose,^94^^,^^95^ increasing dialyzer flux,^94^^,^^96^ and increasing hemodialysis frequency,^97^ have not been found to reduce all-cause or CV mortality. No statistically significant impact on all-cause and cause-specific mortality was revealed by nondialysis interventions (e.g., lowering cholesterol through statin use, use of noncalcium-based phosphate binders [sevelamer] vs. calcium-based binders) in patients undergoing hemodialysis.^85^, ^86^, ^87^, ^88^, ^89^, ^90^, ^91^, ^92^, ^93^, ^94^, ^95^, ^96^, ^97^, ^98^

### Conclusion

CVD is the leading cause of mortality and morbidity in patients with CKD and ESKD. Elevated serum phosphorus levels influence development of CVD through various pathophysiological mechanisms. The current spectrum of treatments aimed at phosphate control is limited to binders that must be taken with meals to avoid systemic absorption of dietary phosphate. Binders have evolved in the last several decades, but achieving serum phosphorus targets remains challenging. Medication side effects, the requirement of multipill regimens, lack of education among patients and providers, and hidden sources of dietary phosphate are some of the factors that contribute to poor phosphate control. As the understanding of intestinal phosphate absorption evolves, newer targets for intervention are being tested. These novel therapies may overcome some issues that result in poor phosphate control and may also decrease pill burden and undesirable side effects. Despite the strong observational association of elevated serum phosphorus levels with CVD, it remains to be seen from randomized controlled trials whether reduction in serum phosphorus levels decreases CV end points in patients with CKD and ESKD.

## Disclosure

JBW reports serving on advisory boards for AstraZeneca, Akebia, Otsuka, Vifor Pharma, Rockwell Medical, Amgen, CSL Behring, GlaxoSmithKline, and Disc Medicine; serving as a consultant for Fibrogen; and serving on the speaker's bureau for AstraZeneca and Akebia. SMD has no relevant affiliations or financial involvement with any organization or entity with a financial interest in or financial conflict with the subject matter or materials discussed in the manuscript apart from those disclosed.
